# Ultrasound-Guided Biopsies: Medium and Large Joints

**DOI:** 10.3389/fmed.2019.00095

**Published:** 2019-05-21

**Authors:** Joaquim Polido-Pereira

**Affiliations:** ^1^Serviço de Reumatologia, Hospital de Santa Maria, Centro Hospitalar e Universitário de Lisboa Norte, Lisbon, Portugal; ^2^Unidade de Investigação em Reumatologia, Faculdade de Medicina, Universidade de Lisboa, Lisbon, Portugal

**Keywords:** synovium, ultrasound, ultrasound guided needle biopsy, ultrasound guided-procedures, synovial membrane

## Abstract

Ultrasound-guided needle synovial biopsies are useful for clinical practice and research in rheumatology. With the emergence of personalized medicine for the treatment of inflammatory rheumatic diseases, it is predicted that this technique will be increasingly used in the near future. Standardized characterization of the technical aspects of ultrasound-guided needle synovial biopsies is needed in order to produce solid evidence on the safety and effectiveness of the technique.

## Introduction

Synovial biopsies have been used for several decades to study synovium. In clinical practice, they have been mostly used to enhance the differential diagnosis in cases of monoarthritis, mostly chronic, being particularly useful for the diagnosis of fastidious infectious agents, infiltrative diseases, and for some selected cases of crystal induced arthropathies. In research, synovial biopsies have been mostly used to clarify the pathogenesis of rheumatic inflammatory diseases, namely rheumatoid arthritis and spondyloarthritis. More recently synovial biopsies are being used to aid in the personalized treatment of rheumatic diseases. Despite all the advances in the treatment of rheumatoid arthritis in recent years, with several biologic agents available, there is still a lack of markers of response to treatment. Synovial membrane studies may aid in this objective (1–4).

There are several ways of collecting synovial tissue, and the four most commonly used nowadays include blind needle, arthroscopic, ultrasound guided with portal and forceps and ultrasound guided needle biopsy (2).

The late 1990s blind needle biopsy was a natural evolution of the older Parker and Pearson's blind biopsy allowing easier collection of synovium (5, 6). Blind needle biopsies are relatively safe, easy to perform with appropriate training but don't allow accurate sampling of the joint (7). Arthroscopic guided biopsies allow direct visualization of the synovium, but, although feasible in medium and large joints (not so in small ones), require operating theater or similar room (8).

With the increasing use of ultrasound in rheumatology two minimally invasive techniques of synovial biopsy were developed: portal and forceps and needle, allowing the study of smaller joints (1, 3). Both techniques seem safe and well-tolerated with appropriate training. Tissue quality/RNA yield is preserved in subsequent biopsies following therapeutic intervention for the ultrasound guided needle biopsies (3). For ultrasound-guided needle biopsies, there seems to be a trend to have a greater yield for large joints, but this aspect lacks confirmation in larger groups of patients (3). The grade of gray scale synovitis seems to be the best predictor of biopsy yield (3).

In a recent multicentre study comparing four different techniques: blind needle, ultrasound guided portal and forceps, ultrasound guided needle biopsy, and arthroscopy biopsy, including biopsies of knees, wrists, ankles, metacarpophalangeal, and proximal interphalangeal joints, it seemed that blind needle biopsy is less reliable than either arthroscopy or ultrasound-guided biopsy, namely regarding the lower amount of gradable synovial tissue. Arthroscopy seems to yield higher amount of tissue but seems only feasible for bigger joints (2).

There is a long time history of performance of blind synovial biopsies, namely through the use of a Parker Person trocar (5). Apart from that there is a significant experience of performing blind, fluoroscopy-guided and, in the past 10–15 years, ultrasound guided joint injections using previously described methods (9).

With this paper we aim to describe our procedures on how to perform an ultrasound guided needle biopsy of the shoulder, elbow, hip, knee, and tibiotalar joints.

## General Technique

The general technique to perform the biopsy of the medium and large joints is an extrapolation of Kelly et al. (3). Video material is available in the website of the author^1^.

Patient positioning is a key for an uneventful procedure. Most of the medium and large biopsies are best performed with the patient supine and the physician seated, but the shoulder joint, by posterior access can be performed with the patient prone and the physician seated or the patient in lateral decubitus and the physician standing. Both patient and physician shall be comfortable in order to safely access the target joint. As in ultrasound guided injections, before the procedure a scan is used to plan the most adequate needle trajectory in order to avoid neurovascular structures (9).

The procedure shall be performed in sterile conditions, in a clean procedures room or in an operating theater. Anesthetic injection (1–3 mL) is performed in the skin and subcutaneous tissue, up to the joint capsule. Afterwards, anesthetic injection of nearly 5 mL in medium joints and 10–15 mL in large joints. The biopsy needle is the Quick Core 16G 10 mm biopsy needle or equivalent. Longer needles are needed to reach deeper joints in obese patients (such as hips or shoulders). A coaxial sheath is not obligatory but aids when the trajectory is long or when the trajectory is close to neurovascular structures. A maximum number of biopsies shall be obtained, without patient discomfort aiming at a total number of 12. At least six to eight shall be used for paraffine embedding and/or frozen (according to local procedures) and the remainder six immersed in RNA-Later for RNA extraction. Six samples per technique shall guarantee good joint sampling, but standardization is required (3). As in ultrasound guided injections an in-plane approach, trying to keep than the needle as parallel as possible to the probe, is the best approach to perform a synovial guided biopsy. If the angle between the probe and needle is superior to 40° the needle is difficult to see, and two possible strategies to enhance needle visualization include either toeing in the probe or doing the puncture site farther away from the probe (9, 10).

### Specific Joints Technique

All procedures described and specific risks are summarized in Table 1.

**Table 1:** Summary of preferential approach for ultrasound guided biopsy of medium and large joints.

**Joint**	**Patient positioning**	**Biopsy approach**	**Specific risks**
Elbow	Patient supine, shoulder slightly abducted and elbow extended and supinated	Anterolateral and proximal approach, through the long extensor carpi radialis and brachioradialis muscles	Muscle rupture or hematoma; Radial nerve lesion
Shoulder	Patient prone or in lateral decubitus with shoulder adducted and with neutral or slight internal rotation	Lateral to medial and posterior approach, through the infraspinatus muscle	Suprascapular nerve or circumflex artery lesion
Tibiotalar	Patient supine with knee with 90° flexion and tibiotalar joint with slight plantar flexion	Anterolateral approach posterior and inferior to the extensor digitorum longus	Tibialis anterior artery, deep peroneal, and superficial peroneal nerve lesion
Knee	Patient supine and knee slightly flexed and supported	Superolateral approach of the suprapatellar pouch	Same as knee joint aspiration
Hip	Patient supine with hip with neutral or slight external rotation and knee extended	Lateral to medial approach, puncture posterior to the sartorius muscle, aiming at the femoral head to neck transition	Lateral femoral cutaneous nerve; femoral neurovascular bundle

#### Elbow Joint

**Figure 1:**
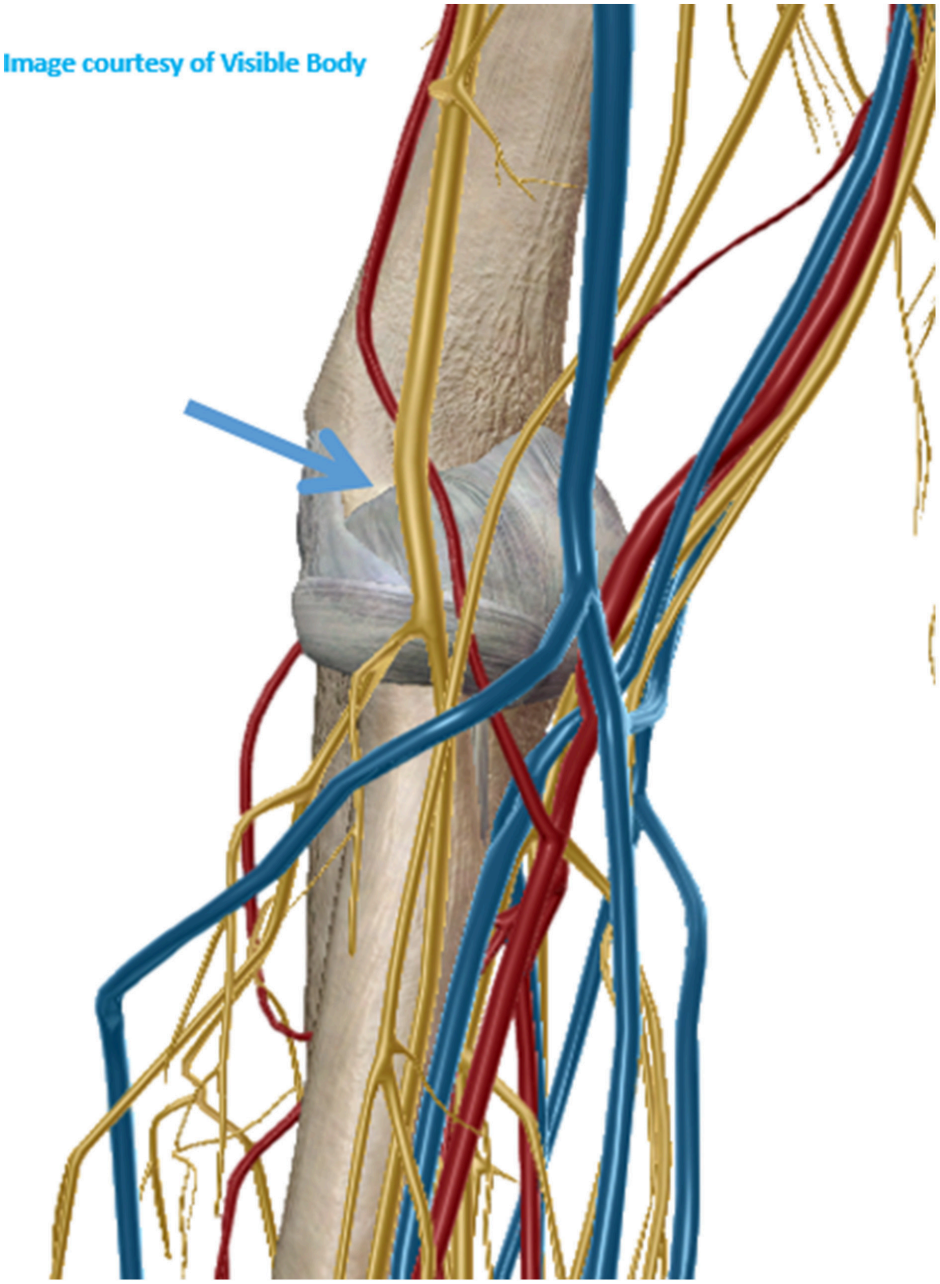
Anterolateral approach for elbow biopsy.

**Figure 2:**
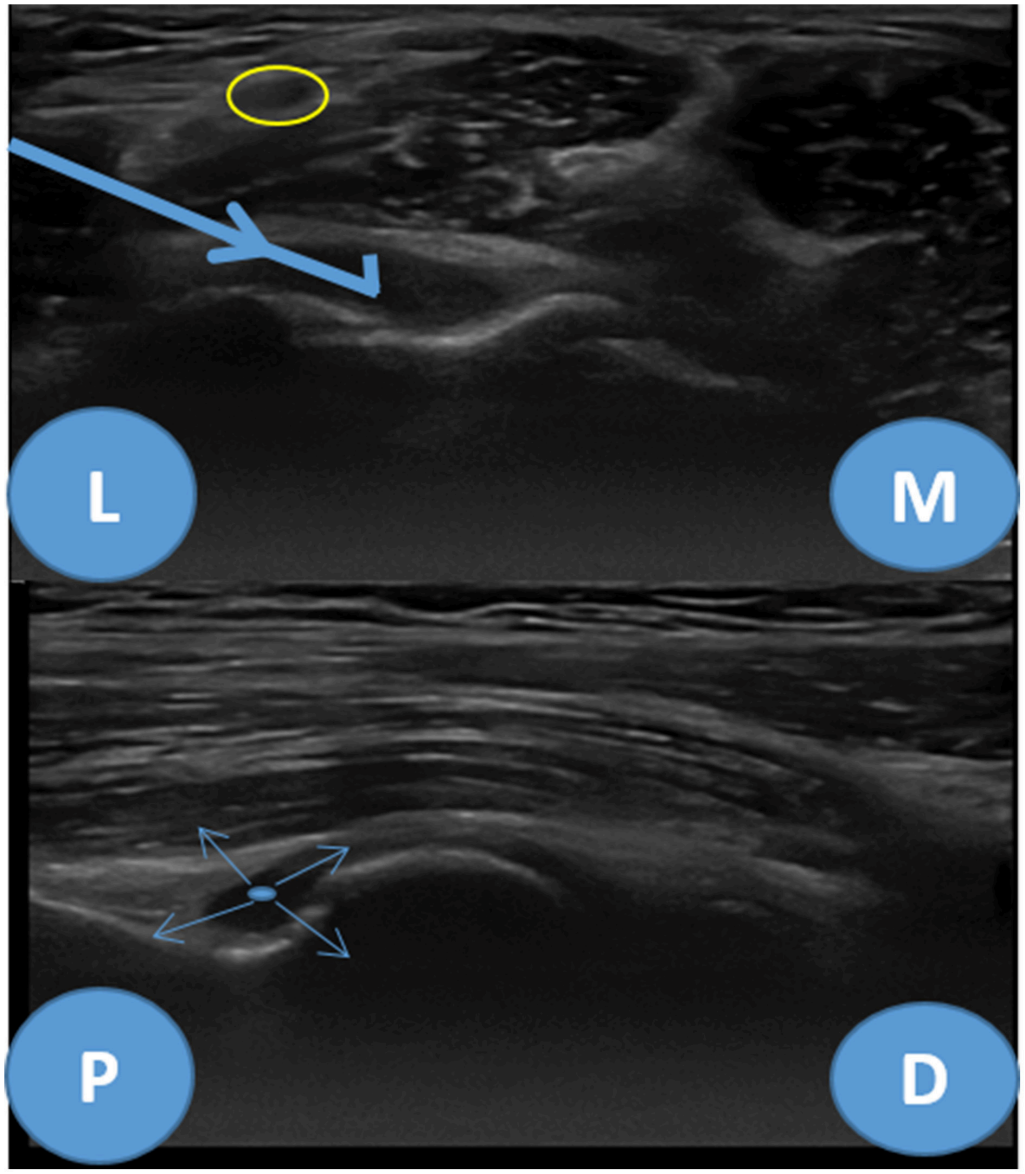
Anterolateral approach for elbow biopsy, ultrasound images. Upper image—transverse view, L, lateral; M, medial. Yellow circle—radial nerve, Bold blue arrow—biopsy needle trajectory. Lower image—longitudinal view, P, proximal; D, distal. Thin blue arrows—needle throw directions.

For ultrasound guided joint injection, the usual approach is laterally through the radiocapitellar joint or posteriorly at the medial or lateral side of the triceps tendon. The medial approach shall be avoided due to the presence of the ulnar nerve that goes through the medial aspect of the triceps tendon (10). However, none of these approaches allows good visualization of the needle throw in ultrasound-guided needle biopsies. One good technique to surpass these difficulties is to approach the elbow joint anterolaterally and proximally, through the long extensor carpi radialis and brachioradialis muscles, posterior to the radial nerve in the radial fossa of the humerus (Figure 1). For this approach, the elbow must be extended and the hand supinated. The proximity of the radial nerve is a caveat and the use of a coaxial sheath may diminish the risk of nerve injury, despite the lack of evidence. After reaching the joint recess the needle throw shall be directed in multiple ways for better sampling (Figure 2).

Patients with limited elbow extension may need to perform the biopsy through the posterior approach with the elbow flexed, laterally to the triceps tendon. The medial approach shall be avoided due to the proximity of the ulnar nerve, as previously referred for the injection.

#### Shoulder Joint

**Figure 3:**
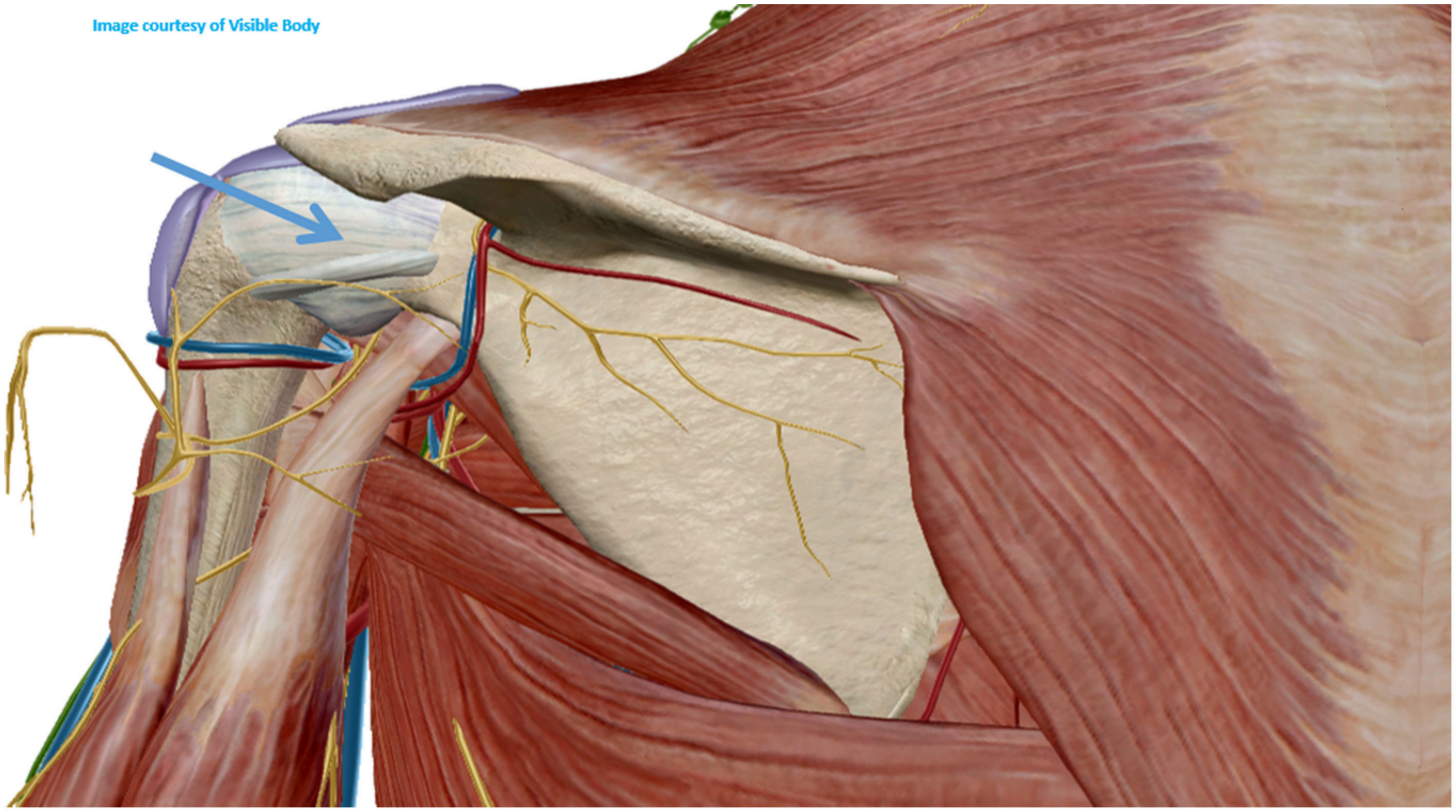
Posterior approach of the shoulder joint.

**Figure 4:**
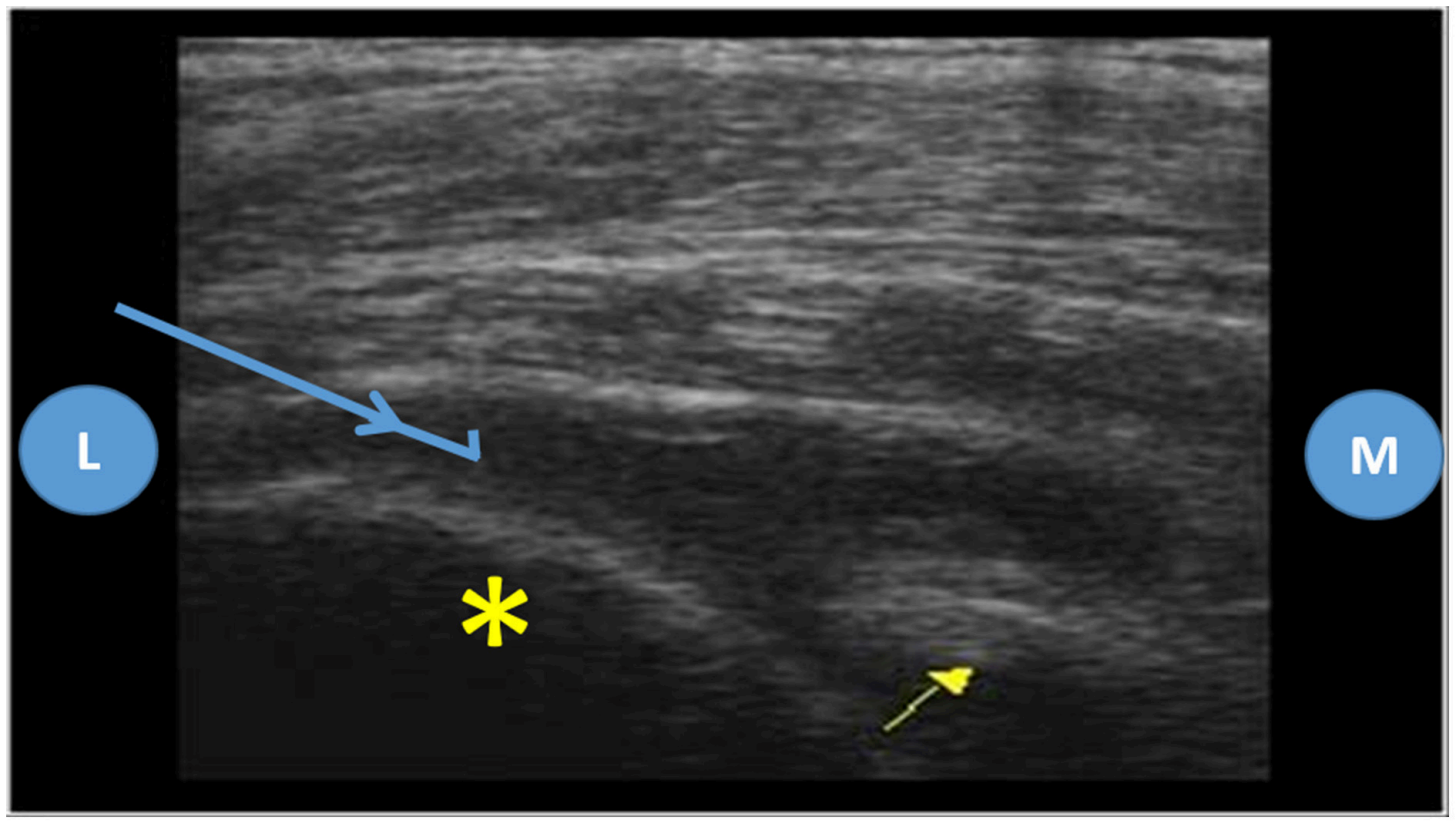
Posterior approach of the shoulder joint, ultrasound images. Probe placed on the long axis of the infraspinatus muscle, L, lateral; M, medial; Yellow asterisk—humeral head; Yellow arrow—glenoid; Blue arrow—biopsy needle trajectory.

Usually the posterior approach is the preferred for the ultrasound guided shoulder injection, and the same applies for the ultrasound guided needle biopsy (9, 11). To perform this approach, the patient shall be either in lateral decubitus or prone, with the shoulder neutral or with slight internal rotation. The transducer shall be aligned with the long axis of the infraspinatus muscle and the lateral to medial approach is usually the better to execute the biopsy due to better placement of the needle throw (more parallel to the probe) (9, 11) (Figures 3, 4).

#### Tibiotalar Joint

The best position to perform either the injection or the biopsy of the tibiotalar joint is with the patient lying supine, with the knee flexed around 90 degrees and the tibiotalar joint in slight plantar flexion.

**Figure 5:**
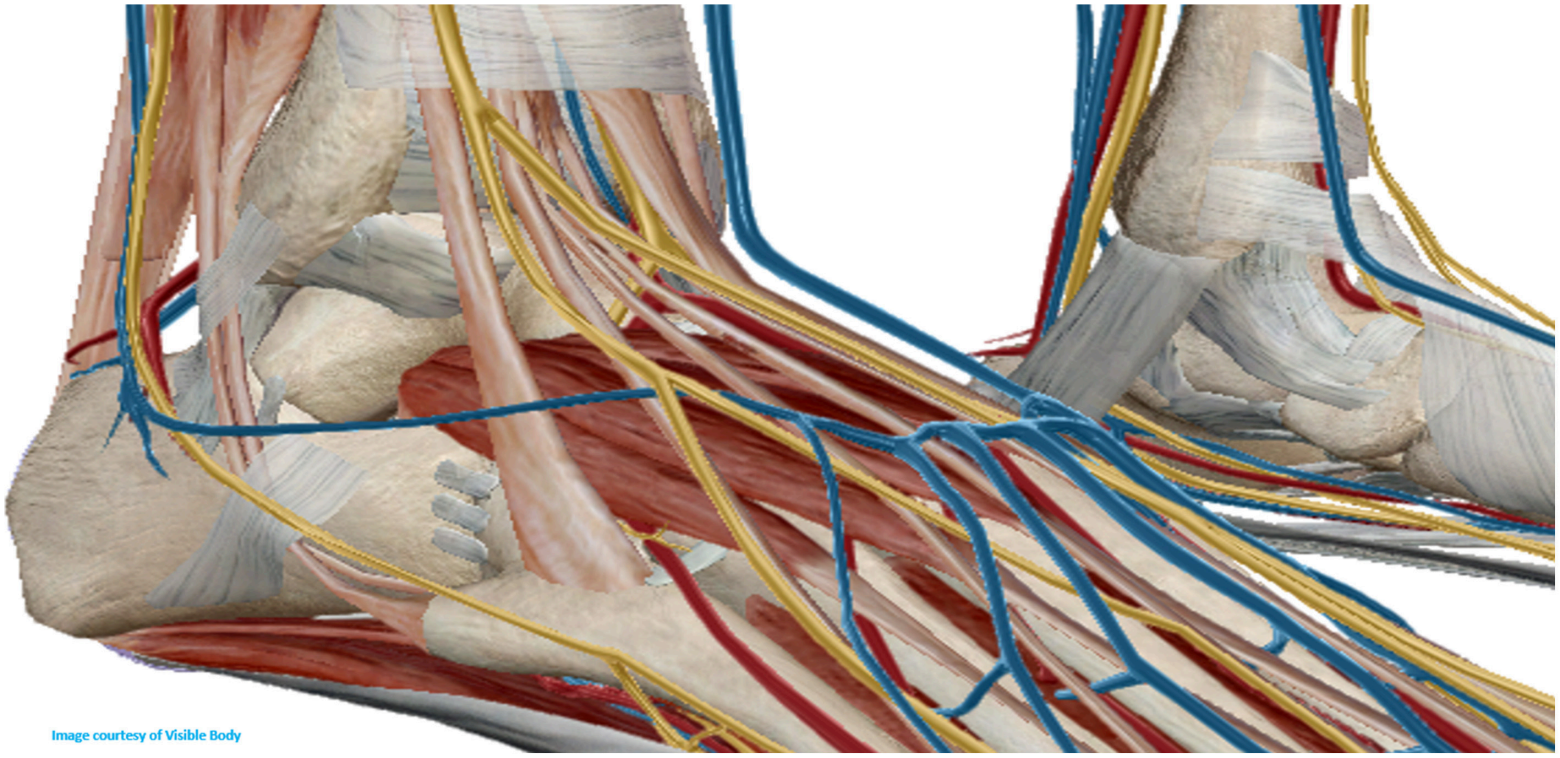
Anterolateral biopsy approach for the tibiotalar joint.

**Figure 6:**
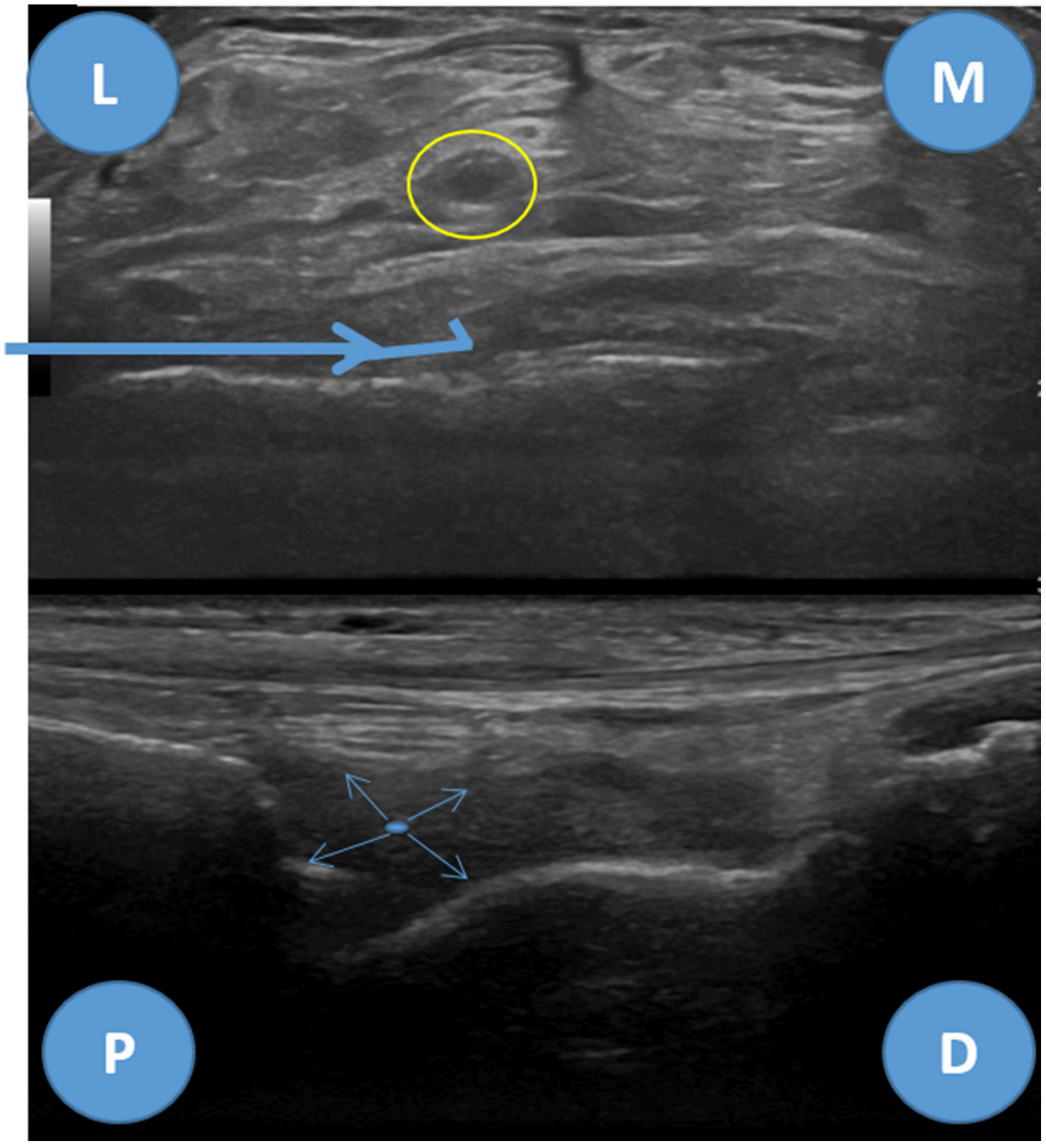
Anterolateral biopsy approach for the tibiotalar joint, ultrasound images. Upper image—transverse view of the tibitalar joint, L, lateral; M, medial. Yellow circle—tibialis posterior artery and deep peroneal nerve, Bold blue arrow—biopsy needle trajectory. Lower image—longitudinal view, P, proximal; D, distal. Thin blue arrows—needle throw directions.

For the injection, usually the preferred approach is between the tibialis anterior and extensor hallucis longus, in order to avoid damage to the anterior tibial artery or to the deep peroneal nerve, which are more lateral (10). However, this approach, being done ultrasound guided, provides poor needle visualization on its long axis, since it is placed almost perpendicular to the probe. One good alternative for the injection, that can be used for the ultrasound guided biopsy, is to perform it with the probe in coronal plane, either medially, just below the tibialis anterior tendon or laterally, just below the extensor digitorum longus (Figures 5, 6).

#### Knee Joint

To perform a knee biopsy the patient is placed supine with the joint slightly flexed and supported, as for the knee joint aspiration (12).

**Figure 7:**
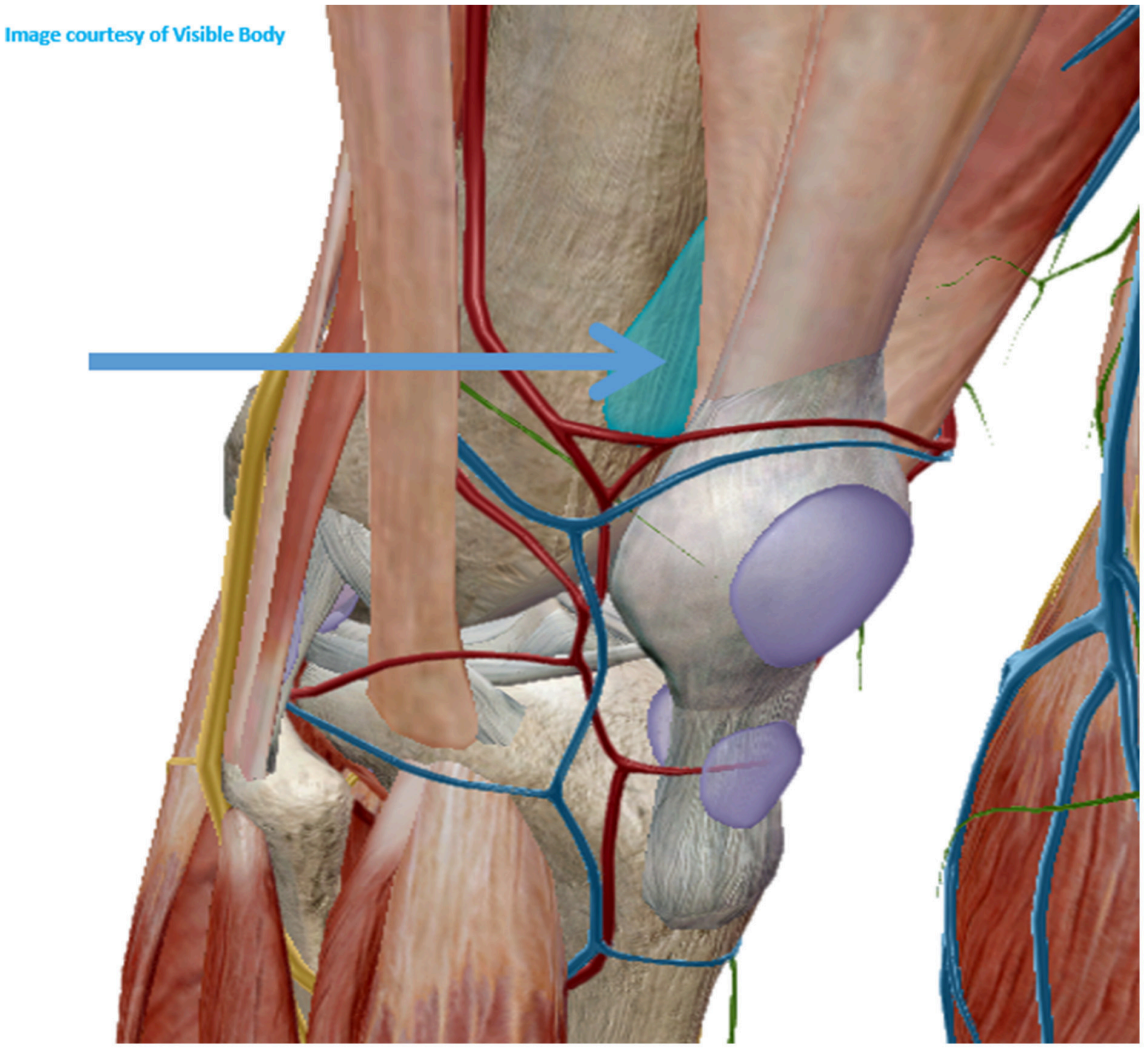
Superolateral approach for knee biopsy.

**Figure 8:**
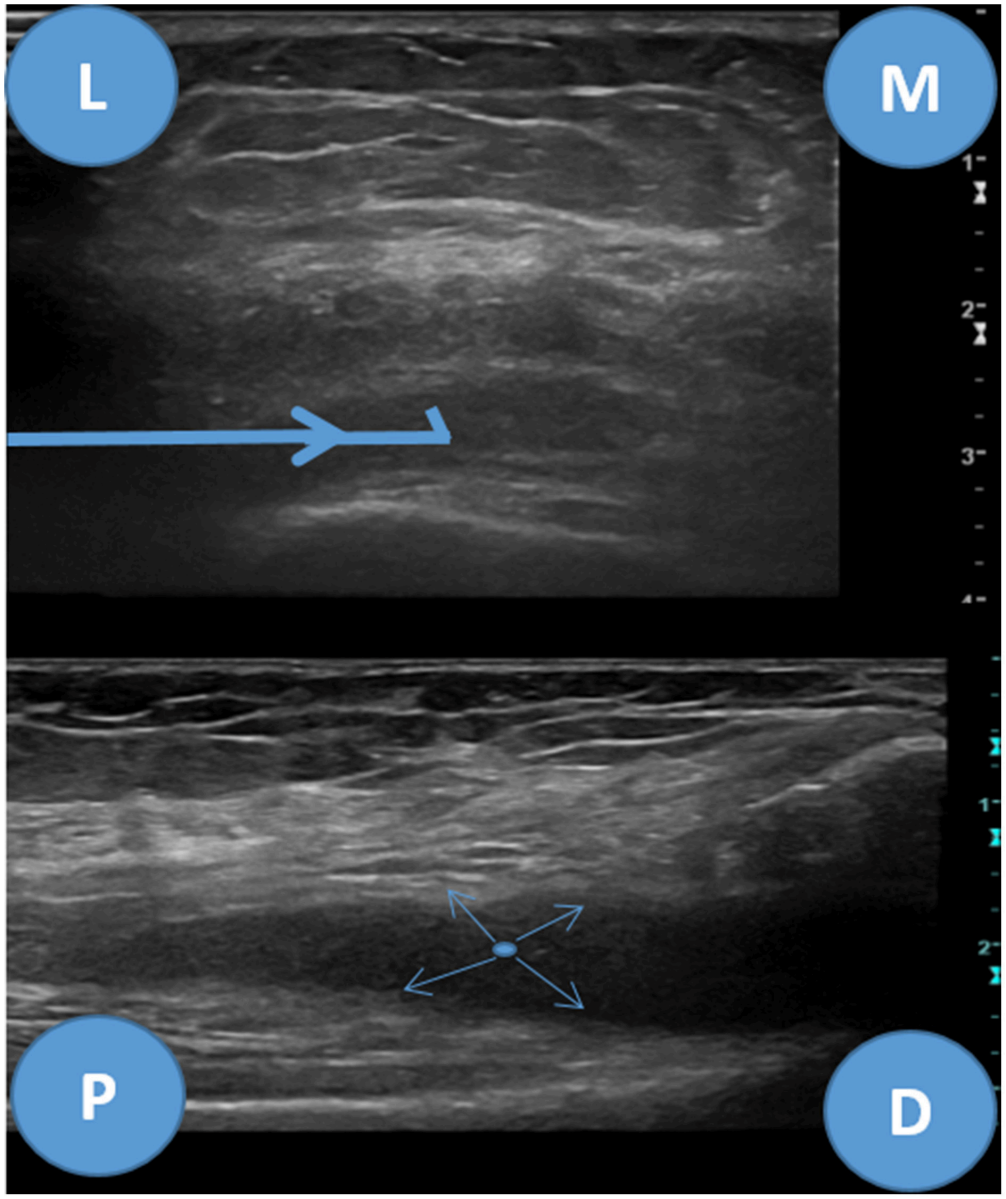
Superolateral approach for knee biopsy, ultrasound images. Upper image—transverse view of the suprapatellar pouch of the knee joint, L, lateral; M, medial. Bold blue arrow—biopsy needle trajectory. Lower image—longitudinal view, P, proximal; D, distal. Thin blue arrows—needle throw directions.

The knee is a large, but quite superficial joint, hence can be easily injected or aspirated, even without ultrasound guidance (13). For blind injections the medial parapatellar approach, 1 cm deep to the patella is easy to perform, but doesn't allow ultrasound guidance because the needle is hidden by the patella. The best approach for the knee ultrasound guided biopsy is therefore the superolateral approach through the suprapatellar pouch, deeply to the quadriceps tendon (Figures 7, 8) (13). Caution shall be taken when doing the puncture in order to avoid the quadriceps tendon, which is quite painful, if punctured (12).

Although easily accessible blindly there is evidence that either injection or biopsy are more precise and better tolerated when ultrasound-guided (2, 12, 14, 15).

#### Hip Joint

**Figure 9:**
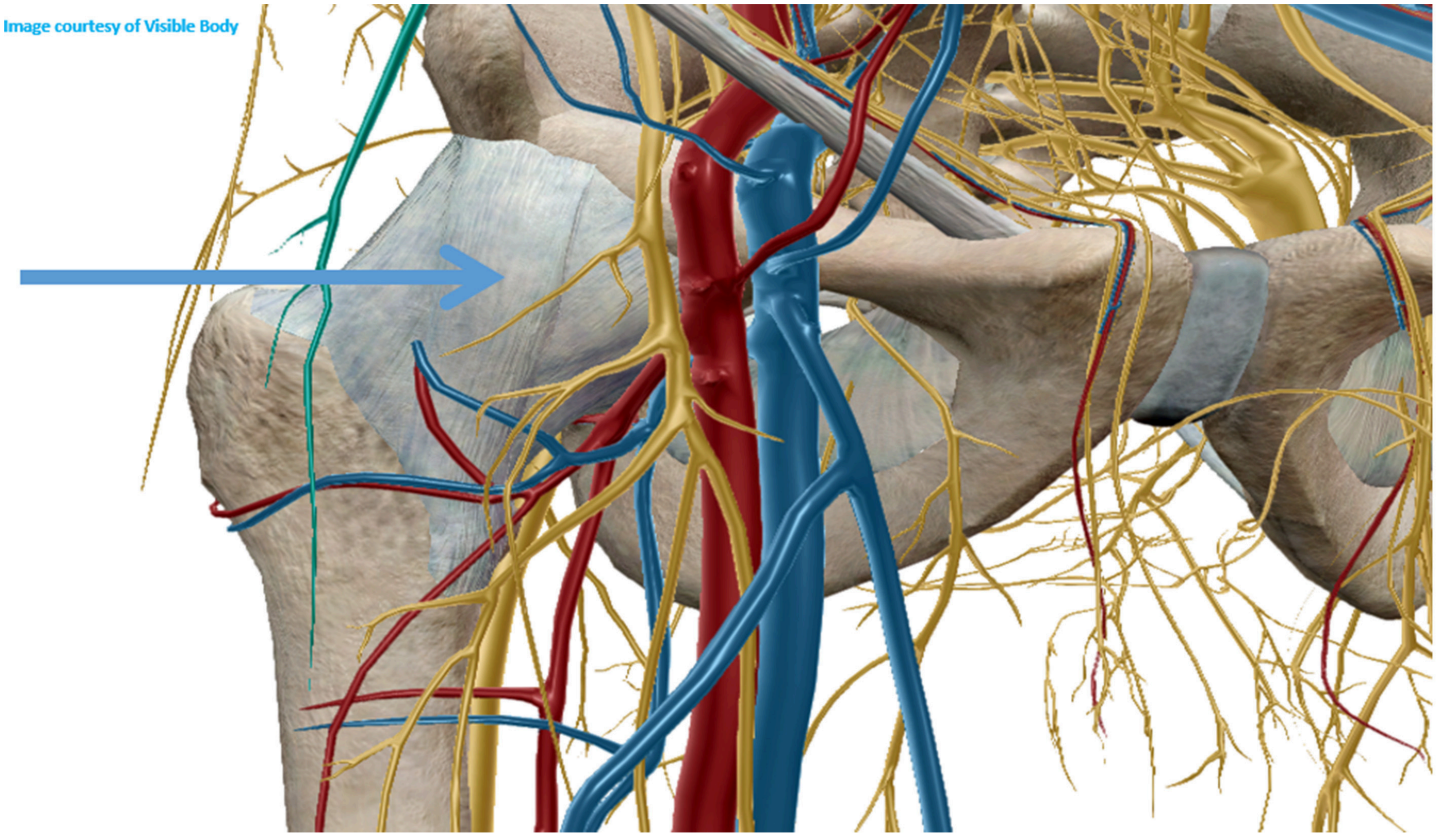
Lateral to medial approach for hip biopsy.

**Figure 10:**
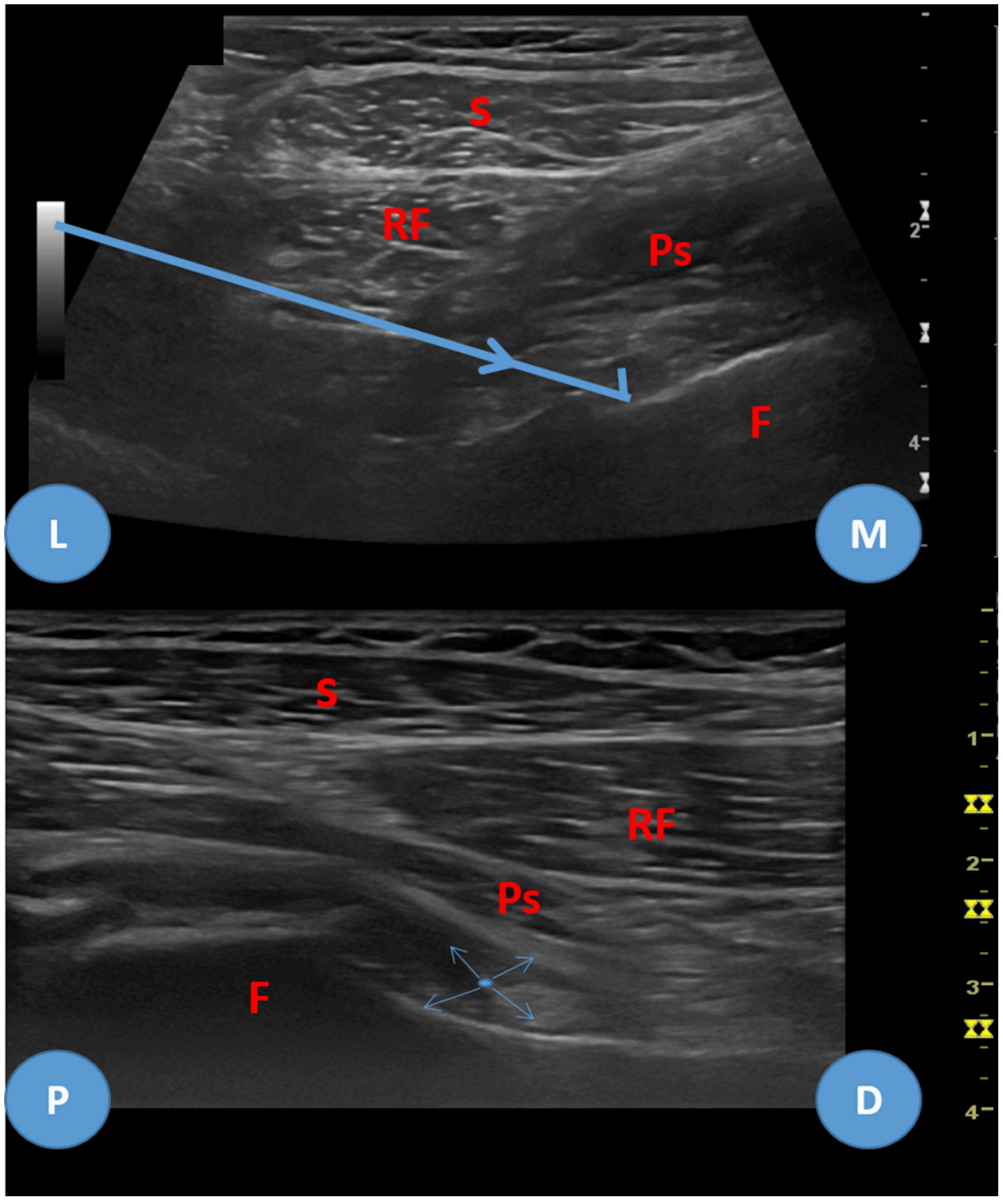
Lateral to medial approach for hip biopsy, ultrasound images. Upper image—transverse-oblique view of the hip joint, L, lateral; M, medial. Bold blue arrow—biopsy needle trajectory. Lower image—longitudinal view, P, proximal; D, distal. Thin blue arrows—needle throw directions. S, sartorius muscle; RF, rectus femoris muscle; Ps, Psoas iliacus muscle and tendon; F, femur.

Although there are descriptions of approaching the hip to inject or aspirate blindly it is a very deep seated joint that shall be injected through imaging guidance (16). Fluoroscopy guided techniques can be performed to target the femoral head, through the sartorius and rectus femoris muscles in a vertical trajectory, or more obliquely, deeply to the sartorius, targeting the femoral neck. These are performed with the patient supine and the hip 10 to 15 degrees internally rotated (17). These techniques are, however, unable to be applied ultrasound-guided. The most commonly used technique to perform an ultrasound guided injection is performed with the patient supine and the hip 20 degrees externally rotated (but according to some authors in neutral position), with the probe longitudinally placed in relation with the femoral neck. The puncture site is usually the more lateral, and the needle shall be placed in perfect alignment with the probe in order to guarantee that the femoral nerve and vessels, which are placed medially, are not harmed. The puncture is done through the rectus femoris and iliopsoas muscles (9, 10, 18). These techniques, albeit suitable for joint injection, due to the fact that the needle trajectory is too steep, don't allow proper needle positioning that permit appropriate ultrasound visualization for the biopsy, nor allow proper biopsy needle throw pressure against the synovium to effectively harvest synovial membrane. One alternative to surpass these limitations is a lateral to medial approach, directed to the femoral head and neck transition, placing the needle more horizontally than the fluoroscopy guided injection technique aiming at the femoral neck described previously by Duc et al. (17) (Figures 9, 10). With this technique, more technically challenging, there is always good needle and femoral neurovascular bundle visualization in relation to the needle (9, 17).

## General Risks

When compared to other biopsies, such as renal biopsy, synovial biopsies (irrespective of the technique used) are very safe when performed by orthopedic surgeons or rheumatologists, and according to some authors lead to <1% of adverse events (19). Up to 25% of patients undergoing ultrasound guided needle biopsy report, at least, mild discomfort or pain and few patients develop a vagal crisis. Joint and skin infections, bleeding and hemarthrosis, post biopsy pain, and neurovascular lesion are rare (3, 20).

### Specific Risks

#### Elbow Joint

Apart from the general risks previously referred the specific risks of the anterolateral approach of the elbow joint are related with the structures that may be injured by the needle trajectory. Long extensor carpi radialis and brachioradialis muscle hematoma or muscle rupture are possible risks as well as lesions of the radial nerve (which can be minimized using a coaxial sheath). There is no evidence on the prevalence of these complications but are expected to be low with practitioners experienced on the use of ultrasound guided procedures.

#### Shoulder Joint

A theoretical risk of this biopsy is the lesion of the suprascapular nerve and circumflex scapular vessels in the spinoglenoid fossa, but that only happens if the needle is placed too medially (9, 11).

#### Tibiotalar Joint

The main structures to avoid regarding the anteromedial or anterolateral pathways for the ultrasound guided biopsy are the anterior tibial artery and the deep peroneal nerve, which are easily seen in the midline (yellow circle in Figure 6) (9). One nerve that can be accidentally punctured in the anterolateral approach is the superficial peroneal nerve, however, in most individuals it is placed superficial to the extensor digitorum longus and, therefore, the biopsy can be safely performed when the needle is placed deeply in relation to this tendon. This nerve, purely sensitive, is a branch of the common peroneal nerve and can be located either in the anterior or the lateral compartment in up to one third of the patients (21–23). In the anteromedial approach, just posterior to the tibialis anterior tendon, care shall be taken in order to avoid the saphenous nerve and the great saphenous vein (21).

#### Knee Joint

Neurovascular structures are far from the superolateral approach hereby described. However, care shall be taken not to puncture the periosteum of the femur or the quadriceps tendon which are significantly painful (12).

#### Hip Joint

One structure than can be harmed in the lateral to medial approach of the hip biopsy is the lateral femoral cutaneous nerve, which is usually located posteriorly to the sartorius and superficially to the rectus femoris and tensor fasciae latae muscles. However, despite in most of the individuals the nerve is located medially to anterior superior iliac spine, there are a lot of variations (it can be located from 6.5 cm medial to 6 cm lateral) (24). For most individuals the nerve courses distally through the flat-filled flat tunnel that lies between the sartorius and tensor fasciae latae muscles, therefore, if the puncture site is through the tensor fasciae latae muscle, there is a low likelihood of harming the nerve (25). The use of a coaxial sheath may diminish this risk.

The most important risk is to harm the femoral neurovascular bundle, if the needle is placed too medially, but this risk is expected to be minor in an experienced practitioner (9, 16, 17).

## Conclusion

Synovial biopsies are of great value even in rheumatology clinical practice nowadays. Ultrasound-guided biopsies are safe, well-tolerated and effective but evidence is lacking. In knee joints the quality of tissue harvested seems superior when compared with wrists, ankles, metacarpophalangeal and proximal interphalangeal joints. There is no evidence of the safety and effectiveness of the procedure for some joints, such as the shoulder or hip. In this paper there is a description of some ways these biopsies can be performed, but technical agreement on how to perform them is needed in order to standardize procedures and to allow the production of solid evidence (2, 3).

## Author Contributions

JP-P is responsible for the conception of the manuscript, including the review of literature, and the individual experience that conducted to the idea.

### Conflict of Interest Statement

The author declares that the research was conducted in the absence of any commercial or financial relationships that could be construed as a potential conflict of interest. The handling Editor declared a shared affiliation, though no other collaboration, with the author JP-P.

## Footnotes

^1^http://www.synovialbiopsy.com
